# Assessment of Behaviors Modeling Aspects of Schizophrenia in *Csmd1* Mutant Mice

**DOI:** 10.1371/journal.pone.0051235

**Published:** 2012-12-17

**Authors:** Margaret G. Distler, Mark D. Opal, Stephanie C. Dulawa, Abraham A. Palmer

**Affiliations:** 1 Department of Pathology, University of Chicago, Chicago, Illinois, United States of America; 2 Committee on Neurobiology, University of Chicago, Chicago, Illinois, United States of America; 3 Department of Psychiatry and Behavioral Neuroscience, University of Chicago, Chicago, Illinois, United States of America; 4 Department of Human Genetics, University of Chicago, Chicago, Illinois, United States of America; Chiba University Center for Forensic Mental Health, Japan

## Abstract

Schizophrenia is a debilitating psychotic disorder that affects up to 1.5% of the population worldwide. Two recent studies in humans identified genome-wide significant associations between schizophrenia and single-nucleotide polymorphisms (SNPs) in an intron of *CSMD1*. The effect of deleting *CSMD1* on mouse behavior is unknown. The present study utilized mice with a mutant *Csmd1* allele in which the first exon had been ablated (KO mice). All *Csmd1* transcripts that included the first exon were absent in the brains of KO mice, but there was persistent expression of at least one other transcript that does not include the first exon. Wild type (WT), heterozygous (HET), and KO mice were assessed using several well-established behavioral paradigms that model aspects of schizophrenia. *Csmd1* KO mice did not differ from wild-type littermates for sensorimotor gating (measured as prepulse inhibition), social interaction, anhedonia (measured by sucrose preference), or sensitivity to the locomotor stimulant effects of the dopaminergic agent *d*-amphetamine. These data demonstrate that loss of *Csmd1* transcripts that include the first exon does not alter multiple well-established behaviors that model aspects of schizophrenia. The SNP most strongly associated with schizophrenia in humans is between exons 3 and 4; therefore, ablation of exon 1 appeared to be a logical animal model. Nevertheless, future studies should consider alternative mouse models including gain-of-function mutations, and loss-of-function mutations that target alternative transcripts of *Csmd1*.

## Introduction

Schizophrenia is a psychotic disorder characterized by positive symptoms (delusions, hallucinations, disorganized speech, and grossly disorganized or catatonic behavior) and negative symptoms (flattened affect, paucity of speech, and reduced motivation) [1]. These debilitating features contribute to profound social and/or occupational dysfunction [1]. The disease has a variable course, ranging from intermittent exacerbations to a progressive and chronic disease. Complete remission almost never occurs [1]. Given its worldwide prevalence of 0.5–1.5%, schizophrenia constitutes a significant public health burden [1].

The exact etiology of schizophrenia is unknown, but human studies have demonstrated significant brain abnormalities, including decreased brain volume, reduced activity of the frontal and temporal lobes, and aberrant neural connectivity [2]. Other hypotheses of the etiology of schizophrenia focus on derangements in neurotransmission. In particular, dopamine is commonly implicated in schizophrenia [2]. Initial support for the dopaminergic hypothesis of schizophrenia came from evidence that drugs that decrease dopaminergic activity reduce psychotic symptoms, while drugs that increase dopaminergic activity produce psychosis [2]. Activity at D2 dopamine receptors is particularly important for mediating psychosis. Although dopaminergic dysfunction in schizophrenic patients has been controversial, there may be an increase in D2 receptor density and alterations in dopamine synthesis and release [2].

It is clear that schizophrenia has a genetic component, as schizophrenia tends to aggregate in families, and there is an approximately three-fold increase in concordance among monozygotic twins compared to dizygotic twins [3]. Nevertheless, schizophrenia is a complex disease, with numerous alleles acting together or independently to affect disease risk [2]. Recently, human genome-wide association studies (GWAS) have been performed in order to identify some of the genetic factors underlying schizophrenia. One such study identified significant associations with several single-nucleotide polymorphisms (SNPs) in *CSMD1*, which encodes CUB and Sushi multiple domains 1 [4]. A subsequent GWAS replicated this finding, demonstrating a significant association between schizophrenia and an intronic SNP in *CSMD1* [5]. Together, these GWAS provide strong rationale for investigating the role of this gene in behavioral mouse models related to schizophrenia.

Several human genetic studies have also identified associations between *CSMD1* and various diseases, including numerous cancers [6]–[15], hypertension [16], metabolic syndrome [17], and psoriasis [18]. *CSMD1* has also been associated with other diseases of the central nervous system (CNS), including multiple sclerosis [19], autism [20], and methamphetamine dependence [21]. Despite these intriguing associations, little is known about *CSMD1*'s function and its mechanism in disease. In fact, only two studies have investigated CSMD1's cellular function. One study demonstrated that CSMD1 has tumor suppressive properties *in vitro* [22], which supports its role in tumorigenesis. A separate study suggested that CSMD1 inhibits the complement cascade [23]. That study also identified high levels of *Csmd1* expression in the CNS of rats, particularly in the neural growth cone during development [23]. Therefore, CSMD1 may be involved in immune processes and neural development.

In the present study, we characterized mice carrying a mutant *Csmd1* allele in which the first exon had been ablated. We tested these mice for behaviors modeling aspects of schizophrenia.

## Methods

### Animals

Constitutive *Csmd1* knock-out (KO) mice were obtained from Taconic Farms (TF0137) and were originally created by Lexicon pharmaceuticals [24]. They were generated by knocking a Neomycin cassette into exon 1 using embryonic stem (ES) cells derived from 129SvEvBrd mice. Live mice used in this study were on a mixed B6:129 background (the exact B6 substrains is not known) and are designated B6;129S5-*Csmd1^tm1Lex^*/Mmucd. Mice from our colony are now available from MMRRC (http://www.mmrrc.org/) with the stock number 32236. Mice for this study were generated by breeding heterozygous (HET) males and females and testing littermate offspring, which should control for the poorly-defined genetic background. All experiments were approved by the IACUC at the University of Chicago.

### *Csmd1* expression

RNA was extracted from whole brains of adult male mice (WT = 4, HET = 7, KO = 3) using the RNeasy kit with DNase digestion (Qiagen). cDNA was generated by reverse transcription (MultiScribe, Applied Biosystems) using oligo dT primers (Invitrogen). cDNA was used as a template in qPCR using SYBR reagents (Applied Biosystems). Each sample was run in duplicate, and the values were averaged for each individual before statistical analysis. Primers targeted *Actb*, exons 1–2 or exon 70 of *Csmd1*. *Csmd1* expression was normalized to *Actb* and reported as fold change versus WT.

### Prepulse inhibition (PPI)

PPI was measured as described previously [25]. Briefly, male and female mice (WT = 15, HET = 58, KO = 21) were moved from the vivarium to a sound-attenuated pre-test room at least 30 minutes prior to the beginning of the test to allow for acclimation to the testing room. At the beginning of the test, each mouse was placed into a cylindrical Plexiglas container (5 cm in diameter), which rested on a platform within a lighted and ventilated chamber (San Diego Instruments, San Diego, CA). Once in the test chamber, mice were presented with 5 minutes of 70-dB white noise, which persisted throughout the remainder of the test. The test consisted of the presentation of 62 trials: a “no stimulus” trial, where no stimulus was presented, a “pulse alone” trial, which consisted of a 40-msec 120 dB burst, and three “prepulse” trials that included a 20-msec prepulse that was either 3, 6, or 12 dB above the 70 dB background noise level followed 100 msec later by a 40-msec, 120-dB pulse. Trials were arranged into four consecutive blocks. The first and fourth blocks consisted of 6 pulse alone trials. The second and third blocks consisted of 25 of the following five trial types—six pulse alone trials, four no stimulus trials, and five of each prepulse trial—in a pseudorandom order. The response to each trial was recorded for 65 ms after the beginning of the 120-dB stimulus or at the beginning of the “no stimulus” trial. The intertrial interval was 9 to 20 s (average 15 s).

The startle response measure (“startle”) was the average startle amplitude for all of the pulse-alone trials and is expressed in arbitrary units. PPI at each intensity was calculated using the formula %PPI = 100%−(100% X [SR_prepulse_/SR_pulse_]), where SR_prepulse_ is the average startle amplitude for prepulse trials, and SR_pulse_ is the average startle amplitude for pulse-alone trials in the second and third testing blocks. The ‘no stimulus’ trials were used to identify technical problems but were not used to calculate any of the phenotypes assessed in this study.

### Social interaction (SI)

Male and female mice (WT = 8, HET = 11, KO = 10) were tested for socialization using a modified SI test [26]. Briefly, each mouse was introduced into a white plastic open field (16″×16″; Accuscan, Columbus, OH) for two consecutive sessions of 5 minutes. During the first session (“target absent”), the open field contained an empty Plexiglas cage with holes (2.5″×4″) positioned at one end of the field. During the second session (“target present”), conditions were identical except that an unfamiliar target mouse was introduced into the Plexiglas cage. Between sessions, the experimental mouse was removed from the open field and placed back into its home cage for 1 min. Tracking data collected from the open field during “target absent” and “target present” conditions were used to determine time spent in an interaction zone (6″×10″) surrounding the Plexiglas cage.

### Sucrose preference (SP)

Anhedonia was assessed using the SP test [27] in the same cohort of mice used for the social interaction test. Male and female mice (WT = 8, HET = 11, KO = 10) were trained to consume a palatable sucrose solution (2%) for 3 days to establish baseline preference levels. During sucrose preference testing and after 18 h of food and water-deprivation, mice were singly housed and presented with two pipettes containing 2% sucrose solution or tap water for 1 h. Pipettes were placed at opposite ends of the home cage. Sucrose preference was calculated by the formula:
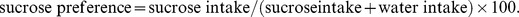



### Locomotor response to *d*-amphetamine

Male and female mice were tested for locomotor response to *d*-amphetamine using a three-day paradigm as described previously [28]. On days 1 and 2, mice were administered an intra-peritoneal (i.p.) injection of vehicle (0.9% saline). Immediately after injection, mice were placed in an open field for 1 hour. On day 3, mice were injected i.p. with 2, 4, or 8 mg/kg *d*-amphetamine HCl (Sigma-Aldrich). Immediately after injection, mice were placed in the open field for 1 hour. Locomotor data were collected and processed using the manufacturer's software (AccuScan Instruments). Group sizes for each dose were as follows: 2 mg/kg: KO = 12; HET = 16, WT = 6; 4 mg/kg: KO = 14, HET = 27, WT = 16, 8 mg/kg: KO = 25, HET = 29, WT = 17.

### Statistical analysis

All statistical tests were performed with StatView (SAS Institute). One-way ANOVAs with genotype as the factor were used except in the following cases. For PPI, we used genotype as a between-groups factor and %PPI as a within-group factor. For SI, we used genotype as a between-groups factor and target presence as a within-group factor. For *d*-amphetamine-induced locomotor activity, we used doses of *d*-amphetamine and genotype as between-groups factors. For the *d*-amphetamine data, we also examined time as a within-groups factor, but it did not interact with genotype, so we collapsed across time and used total distance traveled as the dependent variable. We also considered change from activity at baseline (day 2), but all results were similar, so here we present the data as activity on day 3. Post-hoc tests were performed using Newman–Keuls. In all cases *P*<0.05 was considered statistically significant. In studies that included male and female mice, we did not observe any interactions when sex was included as a factor (e.g. sex*genotype); therefore, both sexes were pooled for all final analyses.

## Results

### *Csmd1* expression

**Figure 1:**
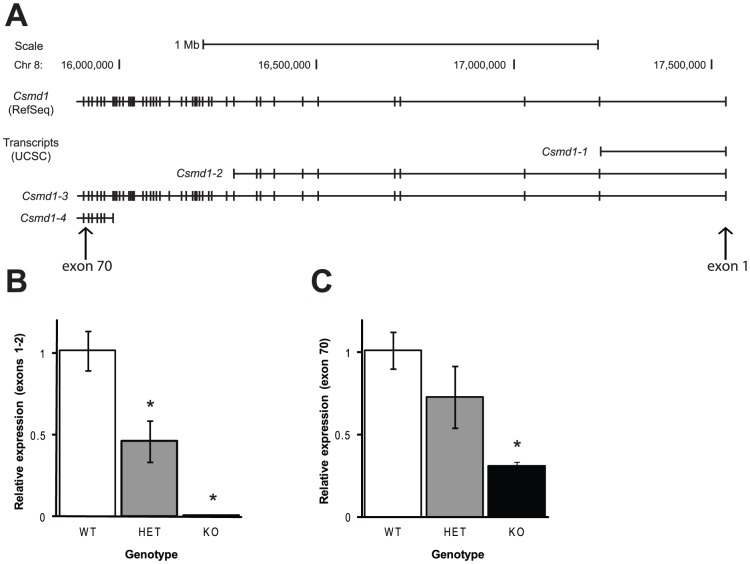
Expression of *Csmd1* transcripts that contain exons 1–2 are lost; however, transcripts containing exon 70 persist, albeit at reduced levels, in KO mice. A) Schematic of the mouse *Csmd1* genomic locus and *Csmd1* transcripts. Exon 1 is on the far right, and exon 70 is on the far left (adapted from UCSC Genome Bioinformatics; http://genome.ucsc.edu). B) Expression of *Csmd1* transcripts that include exons 1–2. C) Expression of *Csmd1* transcripts that include exon 70. * *P*<0.05 versus WT.

*Csmd1* is a large gene that spans over 1.6 Mb and has 70 exons (UCSC Genome Bioinformatics, http://genome.ucsc.edu; Figure 1A). There are four major transcripts (here termed *Csmd1*-1 to *Csmd1*-4), the first three of which begin with exon 1 (Figure 1A). KO mice had a targeted mutation of the first exon of *Csmd1*. We assessed *Csmd1* expression in the whole brains of adult male mice using quantitative real-time PCR (qPCR) with two different primer sets, one specific for exons 1–2 (included in transcripts *Csmd1*-1, -2, and -3) and the other for exon 70 (included in transcripts *Csmd1*-3 and -4). Compared to WT mice, KO mice displayed a 99.4% reduction in expression of exons 1–2 of *Csmd1*, suggesting that the KO allele functioned as expected (F[2,11] = 43.6; *P*<0.0001; Figure 1B). HET mice displayed an approximately 54% reduction in expression of exons 1–2 of *Csmd1* (Figure 1B). For exon 70 of *Csmd1*, KO mice displayed a 70% reduction in expression, and HET mice displayed a 27% reduction in expression compared to WT mice (F[2,11] = 12.2; *P*<0.005; Figure 1C). These results suggest that although expression of *Csmd1*-1, -2, and -3 was ablated, residual expression of the *Csmd1*-4 transcript persisted, as indicated by expression of exon 70. Therefore, KO mice express less than 30% of normal *Csmd1* levels in the brain, all of which appears to come from *Csmd1*-4. While the biological roles of these transcripts are unknown, *Csmd1*-4 may partially compensate for loss of the other three transcripts. Thus, the KO mice used in this study provide insight into the role of *Csmd1* transcripts that include exon 1.

### Behaviors modeling aspects of schizophrenia

Using *Csmd1* KO mice, we assessed several behavioral modalities that are disrupted in schizophrenia: sensorimotor gaiting (as measured using PPI), social interaction, anhedonia (sucrose preference), and sensitivity to a dopaminergic challenge (d-amphetamine induced locomotor response).

### Sensorimotor gating (PPI)

**Figure 2:**
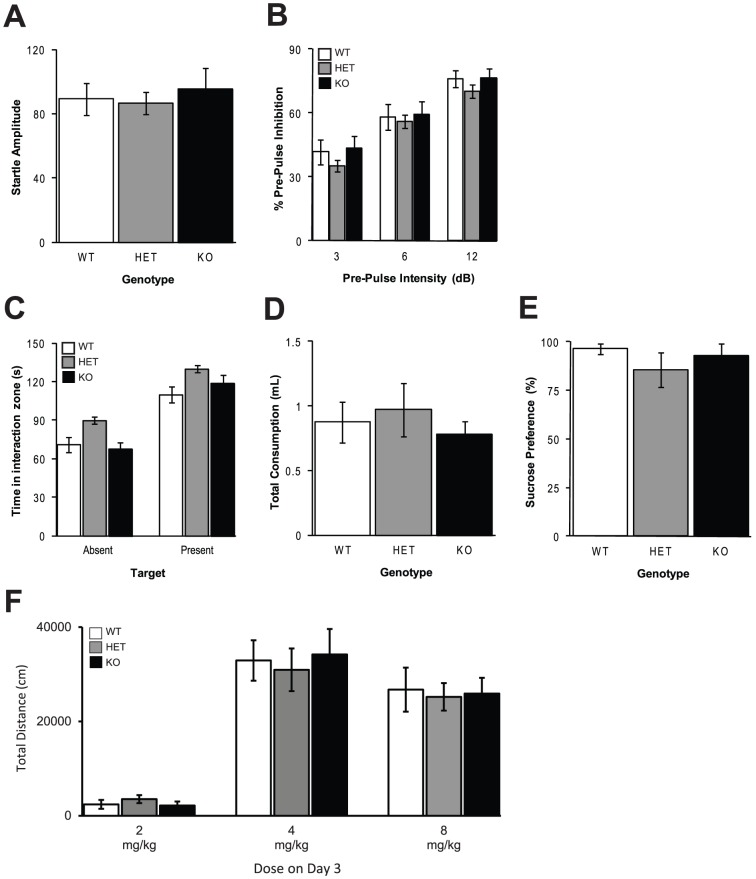
Behavior in *Csmd1* KO mice is normal across multiple behaviors modeling aspects of schizophrenia. A) Startle response did not differ among genotypes. B) There was no significant main effect of genotype on PPI, nor was there a significant interaction between genotype and prepulse intensity. C) During the SI test all mice spent more time in the interaction zone when the target was present versus absent. The total time in the interaction zone did not differ among genotypes, nor was there a significant interaction between genotype and target presence. D) Total consumption of water +2% sucrose did not significantly differ among genotypes in the SP test. E) Sucrose preference did not significantly differ among genotypes. F) Total distance traveled over a one-hour test after treatment with *d*-amphetamine (2, 4, or 8 mg/kg) did not significantly differ among genotypes.

We assessed sensorimotor gating by measuring PPI of the startle response. PPI refers to the ability of a weak, initial stimulus (prepulse) to inhibit the response to a subsequent startling stimulus [29]. Schizophrenic patients display reduced PPI [30], [31]. WT, HET, and KO mice did not differ in startle response (F[2,91] = 0.24; *P* = 0.78; Figure 2A). All mice displayed robust PPI (Figure 2B). As expected, the magnitude of PPI significantly increased as the prepulse intensity increased (F[2,91] = 171.8; *P*<0.001); however, there was no significant main effect of genotype on PPI (F[2,91] = 0.25; *P* = 0.78), nor was there a significant interaction between genotype and prepulse intensity (F[2,91] = 0.94; *P* = 0.44). Thus, *Csmd1* KO mice had normal sensorimotor gating.

### Social interaction (SI) test

Schizophrenic patients display negative symptoms including reduced social behavior, which we modeled using the SI test [30], [31]. WT, HET, and KO mice spent more time exploring the interaction zone when the target (unfamiliar mouse) was present compared to when the target was absent (F[2,26] = 16.8; *P*<0.001; Figure 2C). However, there were no differences among the genotypes in amount of time spent in the interaction zone (F[2,26] = 0.26; *P* = 0.77), nor was there a significant interaction between genotype and target presence (F[2,26] = 0.14; *P* = 0.87). Thus, we did not identify any deficits in social behavior in *Csmd1* KO mice.

### Sucrose Preference (SP) test

Another negative symptom associated with schizophrenia is anhedonia, which we modeled using the SP test [30], [31]. WT, HET, and KO mice consumed both water and a 2% sucrose solution and did not differ in total consumption (F[2,26] = 0.36; *P* = 0.70; Figure 2D). There was no difference among the genotypes in sucrose consumption (F[2,26] = 0.63; *P* = 0.54; Figure 2E), reflecting normal hedonic behavior. Thus, we did not identify any differences in hedonic behavior in *Csmd1* KO mice.

### Psychostimulant response

We then assessed the psychostimulant response to the dopamine-releasing drug *d*-amphetamine. Increased locomotor activation from *d*-amphetamine is thought to model the positive symptoms of schizophrenia [30]. Furthermore, schizophrenic patients have increased dopamine release in response to *d*-amphetamine compared to healthy controls [30]. On the test day (day 3), mice were administered one of three doses of amphetamine (2, 4, or 8 mg/kg, i.p.), and locomotor stimulation was measured. WT, HET, and KO mice showed dose-dependent increases in locomotor activity. While there was a highly significant main effect of dose (F[2,153] = 30.2; *P*<0.0001; Figure 2F), there was no main effect of genotype (F[2,153] = 0.045; *P* = 0.96), nor was there an interaction between genotype and dose (F[2,153] = 0.085; *P* = 0.99). Thus, we did not detect altered sensitivity to *d*-amphetamine in *Csmd1* KO mice.

## Discussion

Human genetic studies have implicated *CSMD1* in schizophrenia [4], [5]. Therefore, we investigated the role of *Csmd1* in behaviors modeling aspects of schizophrenia in mice. To do so, we obtained mice with a null allele of *Csmd1* and assessed them for behaviors using well-established tests that investigate different domains affected by schizophrenia, including sensorimotor gating (PPI), social behavior (SI), hedonic behavior (SP), and psychostimulant response to *d*-amphetamine. We did not observe differences between genotypes in any of these tests. We did not examine learning and memory paradigms, which may be a useful future direction.

Our results suggest that *Csmd1* is not essential for a range of behaviors thought to model key aspects of schizophrenia in mice. However, while the KO mice we studied had reduced expression of three transcripts, *Csmd1-1*, *Csmd1-2*, and *Csmd1-3*, there was residual expression of *Csmd1-4*, because this transcript does not utilize Exon 1 (Figure 1). As such, *Csmd1-4* could have functional redundancy with the other transcripts. Thus, our results suggest that there are no changes in schizophrenia-relevant behaviors in mice lacking transcripts of *Csmd1* that include the first exon. Future studies that investigate the effect of ablation of other *Csmd1* transcripts as well as gain-of-function mutations will help clarify the results of our study.

Is ablation of exon 1-containing transcripts the best mouse model given the available human GWAS data? While it is difficult to fully address this question without knowing the functional differences underlying the human association signal, some speculation is warranted. The expressed transcripts reported as UCSC Genes (http://genome.ucsc.edu) differ between mouse and human. Our study has focused on the four UCSC transcripts in mouse, three of which begin at exon 1. In contrast, while there are also four UCSC *CSMD1* transcripts in human, only one of them begins at exon 1. The SNP most strongly associated with schizophrenia in the human GWAS was rs10503253, which lies in the intron between exons 3 and 4. Thus, ablation of exon 1-containing transcript models loss-of-function mutations that include the linkage disequilibrium block identified by the human GWAS results. The human association signal is almost 1 Mb from the start site of the shorter, non-exon 1-containing human transcripts, which suggests that they are less likely to be related to the observed genetic association at rs10503253. Nevertheless, in the absence of any data describing the functional meaning of the human association, it is not clear how best to model this association in mice. Therefore, future studies of alternative *Csmd1* transcripts in mice may be warranted. Another point to consider is that the first human GWAS study that implicated *CSMD1* actually found stronger evidence implicating *CSMD2* than *CSMD1* [4]. However, that finding was apparently not replicated in a subsequent study [5]. We do not know whether functional redundancy between these two genes exists or whether such redundancy is similar between mice and humans.

In summary, we did not identify any differences between WT, HET, and KO mice for a range of phenotypes that model key aspects of schizophrenia. The fact that we utilized mice that only ablated transcripts including the first exon is an important limitation of our study. Future studies of *Csmd1* in mice should consider other alleles of the gene, including gain-of-function mutations and alterative transcripts. Additional insight into the functional consequences of the SNPs implicated by human GWAS may also help in designing future mouse models.
